# Regulation of *Burkholderia cenocepacia* biofilm formation by RpoN and the c‐di‐GMP effector BerB

**DOI:** 10.1002/mbo3.480

**Published:** 2017-04-16

**Authors:** Mustafa Fazli, Morten Rybtke, Elisabeth Steiner, Elisabeth Weidel, Jens Berthelsen, Julie Groizeleau, Wu Bin, Boo Zhao Zhi, Zhang Yaming, Volkhard Kaever, Michael Givskov, Rolf W. Hartmann, Leo Eberl, Tim Tolker‐Nielsen

**Affiliations:** ^1^ Department of Biology Faculty of Science University of Copenhagen Copenhagen Denmark; ^2^ Costerton Biofilm Center Faculty of Health and Medical Sciences University of Copenhagen Copenhagen Denmark; ^3^ Department of Plant and Microbial Biology University of Zurich Zurich Switzerland; ^4^ Department of Drug Design and Optimization Helmholtz Institute for Pharmaceutical Research Saarland Saarbrücken Germany; ^5^ School of Biological Sciences Nanyang Technological University Singapore Singapore; ^6^ Research Core Unit Metabolomics Institute of Pharmacology Hannover Medical School Hannover Germany; ^7^ Singapore Centre on Environmental Life Sciences Engineering Nanyang Technological University Singapore Singapore

**Keywords:** Biofilm, c‐di‐GMP, *rpoN*, *berB*, *Burkholderia cenocepacia*

## Abstract

Knowledge about the molecular mechanisms that are involved in the regulation of biofilm formation is essential for the development of biofilm‐control measures. It is well established that the nucleotide second messenger cyclic diguanosine monophosphate (c‐di‐GMP) is a positive regulator of biofilm formation in many bacteria, but more knowledge about c‐di‐GMP effectors is needed. We provide evidence that c‐di‐GMP, the alternative sigma factor RpoN (σ54), and the enhancer‐binding protein BerB play a role in biofilm formation of *Burkholderia cenocepacia* by regulating the production of a biofilm‐stabilizing exopolysaccharide. Our findings suggest that BerB binds c‐di‐GMP, and activates RpoN‐dependent transcription of the *berA* gene coding for a c‐di‐GMP‐responsive transcriptional regulator. An increased level of the BerA protein in turn induces the production of biofilm‐stabilizing exopolysaccharide in response to high c‐di‐GMP levels. Our findings imply that the production of biofilm exopolysaccharide in *B. cenocepacia* is regulated through a cascade involving two consecutive transcription events that are both activated by c‐di‐GMP. This type of regulation may allow tight control of the expenditure of cellular resources.

## 1 Introduction

*Burkholderia cenocepacia*, a member of the *Burkholderia cepacia complex* (Bcc), is an opportunistic pathogen causing life‐threatening infections in immune compromised individuals and in patients with cystic fibrosis (Chiarini, Bevinino, Dalmastari, Tabacchioni, & Visca, 2006; Mahenthiralingam, Urban, & Goldberg, 2005). Biofilm formation is a virulence trait of Bcc strains, and has been associated with the persistence of the infection and an increased tolerance to antibiotics compared with planktonic cells (Caraher, Reynolds, Murphy, McClean, & Callaghan, 2007; Sajjan, Keshavjee, & Forstner, 2004). *B. cenocepacia* is frequently isolated from cystic fibrosis patients and is often associated with poor clinical outcome and high mortality resulting from a decline in lung function (Drevinek & Mahenthiralingam, 2010). Therefore, considerable research has been conducted in *B. cenocepacia* to elucidate the mechanisms of biofilm formation (reviewed in Fazli et al., 2014).

It is now widely accepted that the second messenger c‐di‐GMP plays a central role in the regulation of biofilm formation in many bacteria (Boyd & O'Toole, 2012). In general, high intracellular c‐di‐GMP levels induce the production of extracellular biofilm matrix components, whereas low intracellular c‐di‐GMP levels suppress the production of matrix components and promote single cell motility. To regulate these cellular functions, c‐di‐GMP binds to specific effectors, which can be proteins or RNA, and alters their structure or output function (Ryan, Tolker‐Nielsen, & Dow, 2012). The identification and characterization of effectors that sense changes in c‐di‐GMP levels is key to understand how c‐di‐GMP exerts its regulatory function.

We previously provided evidence that c‐di‐GMP controls the production of a biofilm‐stabilizing exopolysaccharide encoded by the *Bcam1330*–*Bcam1341* genes (here designated *bepA*–*L* for *B* *urkholderia* exopolysaccharide locus A to L) in *B. cenocepacia*, through binding to and promoting the activity of the transcriptional regulator Bcam1349 (here designated BerA for *B* *urkholderia* exopolysaccharide regulator *A*) (Fazli, McCarthy, Givskov, Ryan, & Tolker‐Nielsen, 2013; Fazli et al., 2011). In the present report, we provide evidence that c‐di‐GMP also regulates the expression of the *berA* gene. We demonstrate that high c‐di‐GMP levels induce *berA* transcription in a manner dependent on the alternative sigma factor RpoN and the putative bacterial enhancer‐binding protein (bEBP) Bcam1342 (designated BerB for *B* *urkholderia* exopolysaccharide regulator *B*). Furthermore, we demonstrate that BerB binds to c‐di‐GMP and to the *berA* promoter region. However, it appears that c‐di‐GMP does not change the DNA‐binding activity of BerB, suggesting that binding of c‐di‐GMP may have effects on other functions of BerB. Our findings suggest that, upon binding of c‐di‐GMP, BerB activates RpoN‐dependent transcription of *berA*. An increased level of BerA protein in turn promotes Bep exopolysaccharide production, which stabilizes *B. cenocepacia* biofilms. To our knowledge, BerB represents the first example of a bEBP whose activity depends on both c‐di‐GMP and RpoN.

## 2 Experimental procedures

### 2.1 Strains, plasmids, and growth conditions

The bacterial strains and plasmids used in this study are listed in Table 1. All *B. cenocepacia* and *Escherichia coli* strains were grown at 37°C. Luria broth (LB) medium was used for overnight batch cultivation of all bacteria unless otherwise stated. Solid media were prepared with 2% (w/v) agar. A quantity of 80 μg tetracycline (Tet) mL^−1^ (liquid medium), 120 μg Tet mL^−1^ (solid medium), 25 μg gentamicin‐sulfate (Gm) mL^−1^, 100 μg kanamycin‐sulfate (Km) mL^−1^, and 100 μg trimethoprim (Tp) mL^−1^ were used for *B. cenocepacia* strains, and 20 μg Tet mL^−1^, 10 μg Gm mL^−1^, 50 μg Km mL^−1^, 50 μg Tp mL^−1^, 100 μg ampicillin (Amp) mL^−1^, and 25 μg chloramphenicol (Cm) mL^−1^ were used for *E. coli* strains where appropriate.

**Table 1:** Bacterial strains and plasmids used in the study

Strain or plasmid	Relevant characteristic(s)	Reference or source
Strains		
*B. cenocepacia* H111	Clinical isolate from a cystic fibrosis patient	Carlier et al., 2014
*E. coli* DH5α	Used for standard DNA manipulations	Invitrogen
*E. coli* Rosetta DE3 pLysS	Used for protein expression and purification	GE Life Sciences
Plasmids		
pBBR1MCS5	Broad‐host‐range cloning vector; Gm^r^	Kovach et al., 1995
pRK404A	Broad‐host‐range cloning vector; Tet^r^	Ditta et al., 1985
pJN105	Cloning vector; *araC‐*P_BAD_ cassette cloned in pBBR1MCS5; Gm^r^	Newman & Fuqua, 1995
pRK600	Helper plasmid in matings; ori‐ColE1 RK‐mob^+^ RK2‐tra^+^; Cm^r^	Kessler, de Lorenzo, & Timmis, 1992
pmini‐Tn7‐kan‐*gfp*	Delivery vector for mini‐Tn7‐kan‐*gfp*; Kan^r^	Norris, Kang, Wilcox, & Hoang, 2010
pUX‐BF13	Mob^+^ ori‐R6K; helper plasmid providing theTn7 transposition functions in trans; Amp^r^	Bao, Lies, Fu, & Roberts, 1991
pDAI‐SceI‐*pheS*	Cloning vector containing the I‐SceI endonuclease; Tet^r^	Fazli et al., 2015
pGEX‐6P‐2	Glutathione *S*‐transferase (GST) fusion vector; Amp^r^	GE Life Sciences
pDONRPEX18Tp‐SceI‐*pheS*	Gateway compatible gene replacement vector based on SceI and pheS; Tp^r^	Fazli et al., 2015
pDONRPEX18Gm‐SceI‐*pheS*	Gateway compatible gene replacement vector based on SceI and pheS; Gm^r^	Fazli et al., 2015
pYedQ	*E. coli yedQ* (*yhcK*) gene cloned into pRK404A; Tet^r^	Ausmees et al., 2001
pYedQ2	*E. coli yedQ* (*yhcK*) gene cloned into pBBR1MCS5; Gm^r^	Fazli et al., 2015
pBerA	*berA* gene cloned in pBBR1MCS5; Gm^r^	This study
pBerB	*berB* gene cloned in pJN105; Gm^r^	This study
pRpoN	*rpoN* gene cloned in pJN105; Gm^r^	This study
pENTRPEX18Tp‐SceI‐pheS‐*rpoN*	Gene replacement vector for *rpoN*; Tp^r^	This study
pENTRPEX18Gm‐SceI‐pheS‐*berB*	Gene replacement vector for *berB*; Gm^r^	This study
pBepB::*lacZ*	Gene replacement vector used to create *bepB*::*lacZ* fusion	This study
pGEX‐6P‐2::*berA*	*berA* gene fused to GST; Amp^r^	This study
pGEX‐6P‐2::*berB*	*berB* gene fused to GST; Amp^r^	This study
pGEX‐6P‐2::*rpoN*	*rpoN* gene fused to GST; Amp^r^	This study
pBK‐miniTn7‐KmΩSm1	miniTn7 delivery vector, Kan^r^	Koch, Jensen, & Nybroe, 2001
pTn7‐berA‐lacZ	Tn7‐based berA‐lacZ promoter fusion. Native rpoN‐binding site. Kan^r^	This study
pTn7‐berA‐lacZ‐rpoNdel	Tn7‐based berA‐lacZ promoter fusion. Deleted RpoN‐binding site. Kan^r^	This study
pTn7‐berA‐lacZ‐rpoNmut	Tn7‐based berA‐lacZ promoter fusion. Mutated RpoN‐binding site. Kan^r^	This study

### 2.2 Standard molecular methods

Basic molecular and microbiological techniques were according to standard protocols (Sambrook & Russel, 2001). Genomic DNA was isolated using the DNeasy Blood and Tissue Kit (Qiagen), the plasmids were isolated using the QIAprep Spin Miniprep Kit (Qiagen), and the PCR fragments were purified and DNA was extracted from agarose gels using Wizard SV Gel and PCR Clean‐Up System (Promega). Triparental matings from *E. coli* to *B. cenocepacia* were carried out as described previously (Fazli et al., 2011).

### 2.3 Generation of deletion mutants

The deletion mutants investigated in the current study were generated using the method described previously (Fazli, Harrison, Gambino, Givskov, & Tolker‐Nielsen, 2015). The primer sequences used in the procedure are available upon request.

### 2.4 Phenotypic characterization and biofilm formation assays

Colony morphology on solid agar medium, pellicle formation at the air–liquid interface of standing liquid cultures, and flow‐cell biofilm formation assays with *B. cenocepacia* strains were carried out as described previously (Fazli et al., 2013).

### 2.5 Protein production and purification

For production and purification of RpoN, BerA, and BerB proteins, the corresponding genes were fused to the glutathione *S*‐transferase (GST) gene. The genes were PCR amplified and the *rpoN* PCR product was cut with *Eco*RI and *Not*I, and the *berA* and *berB* PCR products were cut with *Bam*HI and *Eco*RI and cloned into the *Eco*RI/*Not*I or *Bam*HI/*Eco*RI digested plasmid pGEX‐6P‐2, respectively. The GST‐fusion constructs were transformed into *E. coli* Rosetta (DE3) pLysS cells for protein expression and purification. The *E. coli* strains containing the GST‐constructs were grown in 1 L of LB at 37°C, and at an OD_600_ of 0.8, the cultures were transferred to an 18°C water bath, and expression was induced with 0.1 mmol/L IPTG for 16 hr. The cells were pelleted by centrifugation at 3,000 *g* for 20 min, solubilized in 40 ml lysis buffer (50 mmol/L HEPES pH 7.5, 150 mmol/L NaCl, 10% glycerol, 5 mmol/L DTT, 0.5% CHAPS, 2% Triton X‐100, 1× Complete Protease inhibitor cocktail [Roche biochemicals]), and lysed using a French press. Soluble protein lysate was cleared by centrifugation at 20,000 *g* for 20 min, before being passed through twice on a column with 3 ml of Glutathione‐Sepharose FF (GE Healthcare). Thereafter, the resin was washed with 20 column volumes of wash buffer (50 mmol/L HEPES pH 7.5, 150 mmol/L NaCl, 10% glycerol, 1 mmol/L DTT, 0.1% CHAPS, 0.4% Triton X‐100, 0.1x Complete Protease inhibitor cocktail), and 10 column volumes of elution buffer (50 mmol/L HEPES pH 7.5, 150 mmol/L NaCl, 10% glycerol, 1 mmol/L DTT). The bound GST‐fusion proteins were then eluted with elution buffer supplemented with 15 mmol/L reduced glutathione, and fractions of 0.5 ml were collected. Fractions were analyzed by SDS‐PAGE, and positive fractions were pooled, dialyzed against elution buffer without glutathione, and protein contents measured.

### 2.6 c‐di‐GMP‐binding assay

Assessment of c‐di‐GMP binding was carried out using the surface plasmon resonance (SPR) assay. SPR binding assays were performed using a Reichert SR7500DC instrument optical biosensor (Reichert Technologies, Depew, NY) and SAD500M sensor chips obtained from XanTec (XanTec Bioanalytics, Düsseldorf, Germany). Scrubber 2 software (Version 2.0c, 2008, BioLogic Software) was used for processing and analyzing the data. Changes in refractive index due to DMSO‐dependent solvent effects were corrected by use of a calibration curve (seven solutions, 4.25–5.75% DMSO in buffer).

Biotinylated c‐di‐GMP was obtained as lyophilized sodium salt with a purity of >95% from Biolog (BIOLOG Life Science Institute, Bremen, Germany). The lyophilized powder was resolved in 1 ml PBS buffer (138 mmol/L NaCl, 5.1 mmol/L KCl, 10.6 mmol/L Na_2_HPO_4_, 1.8 mmol/L KH_2_PO_4_, pH 7.4) to reach a stock concentration of 1 mmol/L. For immobilization, biotinylated c‐di‐GMP was further diluted to obtain a final concentration of approximately 40 μmol/L and injected for 10 min using a flow rate of 5 μl/min at 18°C. After quenching by use of a biotin solution (3 μg/ml), immobilization levels of 1,100 μRIU (sensor chip 1) and 2,600 μRIU (sensor chip 2) were obtained.

The BerA protein (28 kDa) was stored in PBS buffer prior to the SPR analysis at a stock concentration of 17.5 μmol/L. The protein was diluted in PBS buffer first by factor 1:10 and for the following dilutions by factor 1:2, leading to a concentration range of 1.7 μmol/L to 3.3 nmol/L. The BerB protein (51.3 kDa) was stored in HEPES buffer at a final concentration of 39 μmol/L. The protein was diluted in HEPES buffer (10 mmol/L HEPES, 150 mmol/L NaCl, pH 7.4, 0.05% Tween (v/v)) by factor 1:2 to obtain a concentration range of 19.5 μmol/L to 1.1 nmol/L. The binding assays were performed using a constant flow rate of 25 μl/min in PBS buffer for BerA and in HEPES buffer for BerB. The protein dilutions were consecutively injected for 120 s association time and 240 s dissociation time.

### 2.7 Electrophoretic mobility shift assay

A 216‐bp DNA probe spanning −280 to −74 base pairs relative to the translational start of *berA* was generated using PCR. For electrophoretic mobility shift assay (EMSA) analysis, 50 ng of the DNA probe was incubated with purified protein in the presence or absence of 50 μmol/L c‐di‐GMP in binding buffer (50 mmol/L HEPES Ph 7.5, 150 mmol/L NaCl, 10% glycerol, 0.1% NP40, 5 mmol/L MgCl_2_, 1 mmol/L DTT) in a total of 20 μl reaction at room temperature for 40 min. The proteins used were 500 μg RpoN and/or 50 and 500 μg BerB. The DNA‐protein complexes were separated by native 5% PAGE in 0.5 × TBE buffer (45 mmol/L Tris‐borate/1 mmol/L EDTA) in a Hoefer minigel (GE Healthcare) at 150 V for 45 min. Migration of DNA fragments was visualized by incubating the gel for 60 min in SYBR‐GOLD (Thermo Fisher Scientific) diluted 1:10,000 in 0.5xTBE, followed by visualization on a GelDoc (BioRad) system.

### 2.8 Exonuclease III footprinting

A 5′FAM‐tagged 216 bp DNA fragment spanning −280 to −74 base pairs of the *berA* promoter was prepared using the primers 5′FAM‐AGGAGTGTCCGGAAATGAGA and 5′‐ACATTGACAGCGGTTGCG. The 5′FAM‐tagged 216 bp dsDNA was then gel extraction purified to homogeneity. A quantity of 5 μM BerB or 5 μmol/L bovine serum albumin (BSA) was incubated with 100 nmol/L dsDNA at room temperature for 5 min in 20 mmol/L Tris‐HCl, pH 7.4, containing 1.67 mmol/L Mg^2+^ and 150 mmol/L NaCl. The footprinting assay was performed by addition of different amounts (1–10 units) of exonuclease III enzyme to the protein/dsDNA mixtures. After 20 min digestion at room temperature, quenching buffer (final concentration 20 mmol/L Tris‐HCl pH 7.4, 1 mol/L NaCl, 10 mmol/L EDTA) was added to all samples to stop exonuclease III digestion. The remnant fragments of the samples were then analyzed by 4–10% native gradient TBE gel electrophoresis or 10% denaturing urea‐TBE gel electrophoresis. Based on preliminary footprinting results with 100 bp and 10 bp DNA ladders, four 5′FAM‐tagged ssDNA's (78 nt, 104 nt, 126 nt, and 146 nt) with corresponding *berA* promoter sequences were synthesized as the molecular markers to interpret the footprinting results with 10% denaturing Urea‐TBE gels. Finally, the potential BerB binding sites were mapped to the *berA* promoter region.

### 2.9 Quantitative real‐time PCR

RNA was isolated from biofilm grown *B. cenocepacia* strains on membrane filters placed on AB medium supplemented with 2% agar (w/v), 0.2% glucose (w/v), 0.1% casaminoacids (w/v) for 48 hr using the RNeasy Protect Bacteria Mini Kit (Qiagen), and it was DNase treated using the Turbo DNA‐free kit (Ambion) according to manufacturers’ instructions. cDNA synthesis and quantitative RT‐PCR analysis were carried out using the Qscript 1‐Step SYBR green qRT‐PCR kit (Quanta Biosciences) according to manufacturer's instructions. As a control, quantitative RT‐PCR was similarly applied to analyze the expression of the *gyrB* gene. The relative expression levels of the target genes were calculated using the threshold cycle (2^−ΔΔCt^) method (Livak & Schmittgen, 2001).

### 2.10 lacZ promoter fusion constructions and β‐galactosidase activity assays

The *B. cenocepacia* H111 *bepB*::*lacZ* reporter strain, which carries a *lacZ* gene together with a ribosome‐binding site (RBS) downstream of the *bepB* gene, was used to assay the expression of *bepB*. In‐frame deletions of target genes were introduced into this reporter strain using the homing endonuclease I‐SceI‐based method described by Flannagan, Linn, and Valvano (2008). C‐di‐GMP‐overproducing strains were constructed by transformation with pYedQ.

Tn7‐based *berA‐lacZ* promoter fusions with different alterations of the putative RpoN‐binding site were used to investigate the role of the binding site for transcriptional activity. They were constructed in the following way: Initially, the *berA* promoter region spanning nucleotides −689 to −41 relative to the *berA* translational start site was amplified using primers introducing XhoI and HindIII overhangs (sequences are available upon request). The promoter regions with alterations of the putative RpoN‐binding site were amplified by SOE‐PCR using primers introducing the alterations. The amplified promoter regions were subsequently cloned by XhoI/HindIII digestion and ligation into the broad‐host‐range *lacZ*‐based promoter probe vector pSU11 (Malott et al., 2009). Finally, blunt‐ended XbaI‐digested fragments from the pSU11‐based vectors encompassing the *berA::lacZ* fusion preceded by a transcriptional terminator were inserted into the blunt‐ended NotI digested Tn7‐delivery vector pBK‐miniTn7‐KmΩSm1. The resulting pTn7‐*berA*‐*lacZ* vectors were introduced into the *B. cenocepocia* genome through four‐parental mating as previously described (Fazli et al., 2013). C‐di‐GMP‐overproducing strains were constructed by transformation with pYedQ2.

For β‐galactosidase activity assays, the strains to be tested were grown overnight in LB broth at 37°C with shaking and used to inoculate 20 ml of AB medium supplemented with 0.2% glucose (w/v) and 0.1% casamino acids (w/v) to an OD_600_ of 0.05. The cell cultures were incubated with shaking for 24 hr to 48 hr at 37°C. β‐Galactosidase activity was measured as described by Stachel, An, Flores, and Nester (1985) with minor modifications. Briefly, 50–200 μl of the cell culture was harvested by centrifugation and resuspended in 500 μl Z‐buffer. After addition of 25 μl of CHCl_3_ and 25 μl of 0.05% SDS (w/v), the cell suspension was vortexed for 10 s and then incubated at 30°C for 15 min. The reaction was started by adding 200 μl of ONPG (4 mg/ml) and incubated at 30°C. The reaction was stopped by addition of 250 μl of 1 mol/L Na_2_CO_3_. The cell debris was removed by centrifugation and the absorbance was recorded at 420 nm and 550 nm. β‐Galactosidase activity was calculated as Miller Units, using the formula Miller Units = 1000x[OD_420_–(1.75xOD_550_)]/(time_[min]_xV_[ml]_xOD_600_).

## 3 Results

### 3.1 RpoN is important for biofilm formation by *B. cenocepacia*

**Figure 1:**
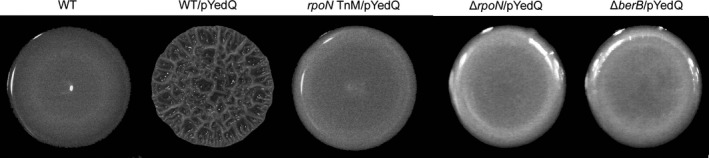
Colony morphology on AB agar medium of the wild‐type (WT) strain, the WT strain carrying pYedQ (WT/pYedQ), the representative *rpoN* transposon‐mutant strain carrying pYedQ (*rpoN* TnM/pYedQ), and the Δ*rpoN* and Δ*berB* strains carrying pYedQ (Δ*rpoN*/pYedQ and Δ*berB*/pYedQ)

We previously showed that high cellular c‐di‐GMP levels in *B. cenocepacia*, achieved through ectopic expression of the *E. coli* diguanylate cyclase YedQ, lead to wrinkled colony formation on solid medium (Fazli et al., 2011). This phenotype depends on the ability of bacteria to produce extracellular components, which often are important constituents of their biofilm matrix (Branda, Vik, Friedman, & Kolter, 2005; Friedman & Kolter, 2004a,b; Rainey & Travisano, 1998; Spiers, Bohannon, Gehrig, & Rainey, 2003; Spiers, Kahn, Bohannon, Travisano, & Rainey, 2002). We exploited the wrinkled colony morphology of the *B. cenocepacia* strain with high c‐di‐GMP levels, and performed a screen of a transposon mutant library for smooth colony formers to identify downstream components of the c‐di‐GMP signaling cascade. Three of the mutants that did not form wrinkled colonies carried a transposon insertion in the *rpoN* gene. Figure 1 shows the colony morphology of the wild‐type *B. cenocepacia*, and a wild‐type strain harboring the plasmid pYedQ causing high c‐di‐GMP levels due to overexpression of the YedQ diguanylate cyclase, and one of the *rpoN* transposon mutants harboring pYedQ.

**Figure 2:**
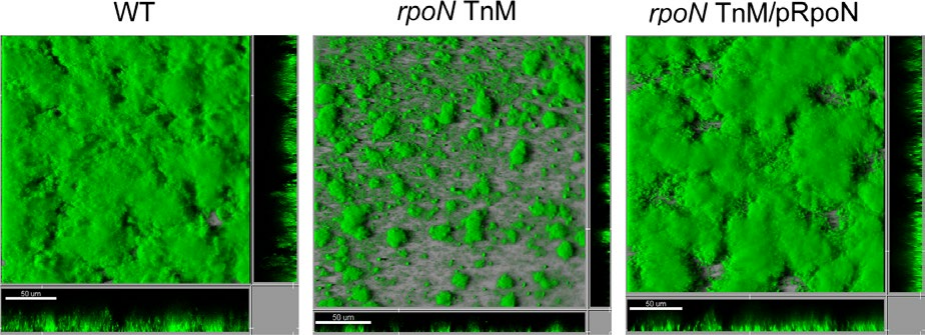
Confocal laser scanning microscope images (CLSM) of SYTO9‐stained, 2‐day‐old flow‐cell biofilms formed by the wild‐type strain, the representative *rpoN* transposon‐ mutant strain (*rpoN* TnM) and the complemented *rpoN* TnM (*rpoN* TnM/pRpoN) strain. The central images show top‐down shadow projection views, whereas the flanking images show vertical sections through the biofilms. The scale bars correspond to 50 μm

The smooth colony morphology of the pYedQ‐containing *rpoN* transposon mutants suggested that they are possibly defective in producing biofilm matrix components. To investigate the role of *rpoN* in *B. cenocepacia* biofilm formation without overexpression of a diguanylate cyclase, we cured the transposon mutants from the pYedQ plasmid and tested them in a flow‐cell biofilm system. The *rpoN* transposon mutants were not severely affected in initial surface attachment, as they were able to colonize the glass surface and form small microcolonies. However, they were markedly impaired in biofilm maturation compared with the wild‐type strain (Figure 2). We were able to restore the biofilm formation ability of the mutants to wild‐type levels by complementation with an intact copy of *rpoN* on a plasmid (Figure 2). We generated a deletion mutant of *rpoN* to confirm the results obtained with the *rpoN* transposon mutants. As expected, the Δ*rpoN* strain was also impaired in biofilm formation in flow cells, and the biofilm formation defect was rescued when it was complemented with an intact copy of *rpoN* on a plasmid (data not shown). Moreover, unlike the wild‐type strain, the Δ*rpoN* strain did not form wrinkled colonies when transformed with the pYedQ plasmid (Figure 1).

### 3.2 The promoter region of *berA* has putative RpoN‐binding sites

We carried out a bioinformatics analysis using the publicly available Regulatory Sequence Analysis Tool (Medina‐Rivera et al., 2015; http://prokaryotes.rsat.eu) to identify RpoN‐dependent genes in *B. cenocepacia* H111. Based on the results obtained from the biofilm experiments, we hypothesized that RpoN might regulate production of biofilm matrix components in *B. cenocepacia*. Hence, we chose to analyze the upstream intergenic sequences of the genes predicted to be involved in production of exopolysaccharides, lipopolysaccharides, surface appendages, and adhesins in *B. cenocepacia* (Holden et al., 2009). In the analysis, we used the position‐specific weight matrix built by Dombrecht, Marchal, Vanderleyden, and Michiels (2002) based on a set of 186 characterized RpoN‐binding sites from 44 different bacterial species as input (Barrios, Valderrama, & Morett, 1999). We found that the *berA* gene is preceded by a putative RpoN‐binding site, ctatTGGCACGTAAATCGCTtatt, located at −196 to −173 base pairs relative to the predicted translational start of *berA*. It contains the highly conserved GG and GC nucleotides positioned at the −24 and −12 elements, respectively (Barrios et al., 1999). This finding supported our hypothesis because *berA* encodes for a c‐di‐GMP‐binding protein (Fazli et al., 2011), which regulates the transcription of the *bep* exopolysaccharide genes in a c‐di‐GMP‐dependent manner (Fazli et al., 2013). We also identified putative −35 and −10 promoter elements located upstream of the RpoN‐binding site, which can be recognized by the house keeping sigma factor RpoD. Further analysis of the upstream sequences of *berA* orthologs from other sequenced *B. cenocepacia* strains revealed that the RpoN‐binding site preceding *berA* is conserved and aligns with the recently derived consensus sequence for the RpoN‐dependent promoters in *B. cenocepacia* H111 (data not shown) (Lardi et al., 2015). Together these results suggest that RpoN plays a role in *B. cenocepacia* biofilm formation by positively regulating expression of the *berA* gene.

### 3.3 BerB, a putative bacterial enhancer‐binding protein, is important for wrinkled colony formation, and the stability of *B. cenocepacia* biofilms

Sequence analysis of the genomic region adjacent to the *bep* exopolysaccharide gene cluster and *berA* revealed the presence of a gene locus, I35_5193, coding for a putative RpoN‐interacting transcriptional regulator. Due to its role in regulating expression of the *bep* genes described below, we designated the protein encoded by the I35_5193 locus BerB (*B* *urkholderia* exopolysaccharide regulator B). Such regulators, called bEBP, typically bind to the DNA sites located upstream of RpoN‐dependent promoters, make contact with the DNA‐bound RpoN‐RNA polymerase closed complex through DNA looping and remodel the closed complex into a transcriptionally active open complex using the energy derived from ATP hydrolysis (Bush & Dixon, 2012). The analysis of the BerB amino acid sequence using NCBI's conserved domain database revealed that BerB contains a central AAA+‐type ATPase domain with the signature GAFTGA motif located at residues 208–213 and a C‐terminal DNA‐binding domain, but lacks an N‐terminal regulatory‐sensory input domain. The presence of the GAFTGA motif, which is almost invariant in bEBPs and is essential for their interaction with RpoN (Zhang et al., 2002), suggests that BerB is a bEBP that interacts with RpoN. A map of the *berA*/*berB* region on the *B. cenocepacia* genome is shown in Figure S1.

**Figure 3:**
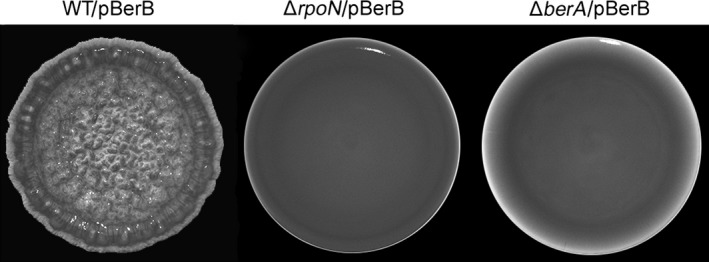
Colony morphology on AB agar medium of the wild‐type, Δ*rpoN* and Δ*berA* strains harboring pBerB

We hypothesized that BerB together with RpoN may have a role in regulating expression of *berA* in *B. cenocepacia*. Accordingly, we generated a Δ*berB* deletion mutant, transformed the Δ*berB* strain with the pYedQ plasmid, and investigated the morphology of colonies formed by the Δ*berB*/pYedQ strain on agar medium. Unlike the pYedQ‐containing wild‐type strain, the pYedQ‐containing Δ*berB*‐mutant strain did not form wrinkled colonies on solid medium (Figure 1). On the contrary, when we overexpressed the *berB* gene from a multicopy plasmid in the wild‐type *B. cenocepacia* strain, the bacteria formed wrinkled colonies on solid medium (Figure 3). The induction of wrinkled colony formation by BerB was dependent on the presence of both the *rpoN* and *berA* gene (Figure 3). These results indicate that *berB* plays a role in the regulation of the *bep* exopolysaccharide genes in a manner dependent on RpoN, BerA, and c‐di‐GMP.

**Figure 4:**
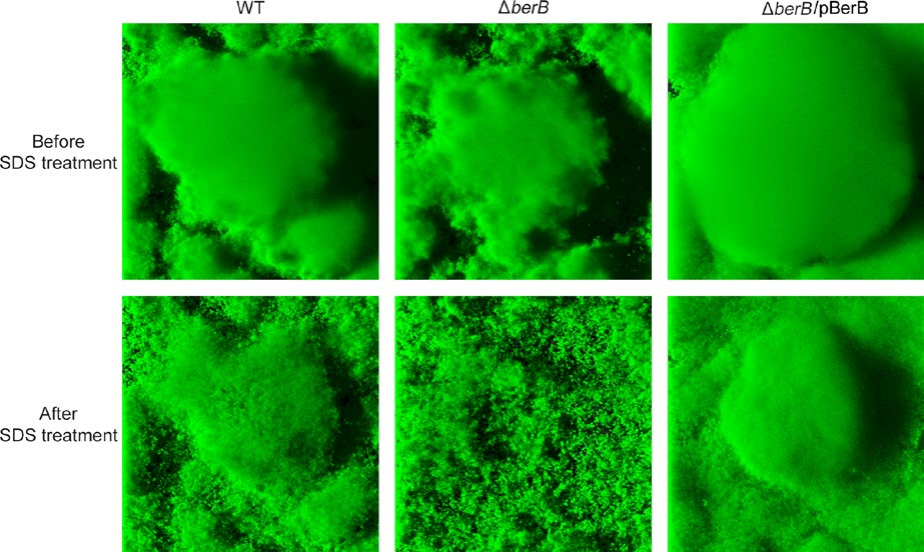
CLSM images of 2‐day‐old flow‐cell biofilms formed by the wild‐type strain, and the Δ*berB* and the complemented Δ*berB* (Δ*berB*/pBerB) strains before and after sodium dodecyl sulfate treatment

We also tested the ability of the Δ*berB* mutant to form biofilm in flow cells. Similar to what we have reported earlier for a Δ*berA* strain (Fazli et al., 2011), the Δ*berB* strain had a defect in biofilm formation, forming very fragile biofilms with increased sensitivity to SDS treatment (Figure 4). This defect was rescued when the Δ*berB* strain was complemented with an intact copy of *berB* on a plasmid (Figure 4).

### 3.4 Mutations of *rpoN* and *berB* do not affect c‐di‐GMP levels in *B. cenocepacia*

One explanation for the negative effects of the *rpoN* and *berB* mutations on wrinkled colony and biofilm formation could be that the mutations affect the c‐di‐GMP level in the bacteria, which in turn influence cellular functions involved in biofilm formation. To test this, we measured c‐di‐GMP concentrations in the wild‐type and mutant strains growing in biofilms or in liquid cultures. Consistent with the previously published results (Fazli et al., 2011), we observed a substantial increase in the cellular c‐di‐GMP levels in the pYedQ‐containing wild‐type strain compared with the wild‐type strain under both growth conditions (Figure S2). We found that the deletion of neither *rpoN* nor *berB* substantially affected the elevated cellular c‐di‐GMP levels caused by ectopic expression of the diguanylate cyclase YedQ. Both the pYedQ‐containing Δ*rpoN* and Δ*berB* strains had increased levels of c‐di‐GMP similar to the pYedQ‐containing wild‐type strain (Figure S2), indicating that the biofilm formation defects caused by the *rpoN* and *berB* mutations are not due to an effect on c‐di‐GMP levels.

### 3.5 RpoN and BerB promote the transcription of *berA* in response to high c‐di‐GMP levels

**Figure 5:**
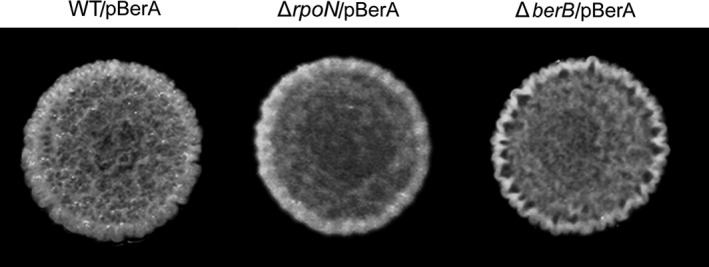
Colony morphology on AB agar medium of the wild‐type, Δ*rpoN*, and Δ*berB* strains harboring pBerA

**Figure 6:**
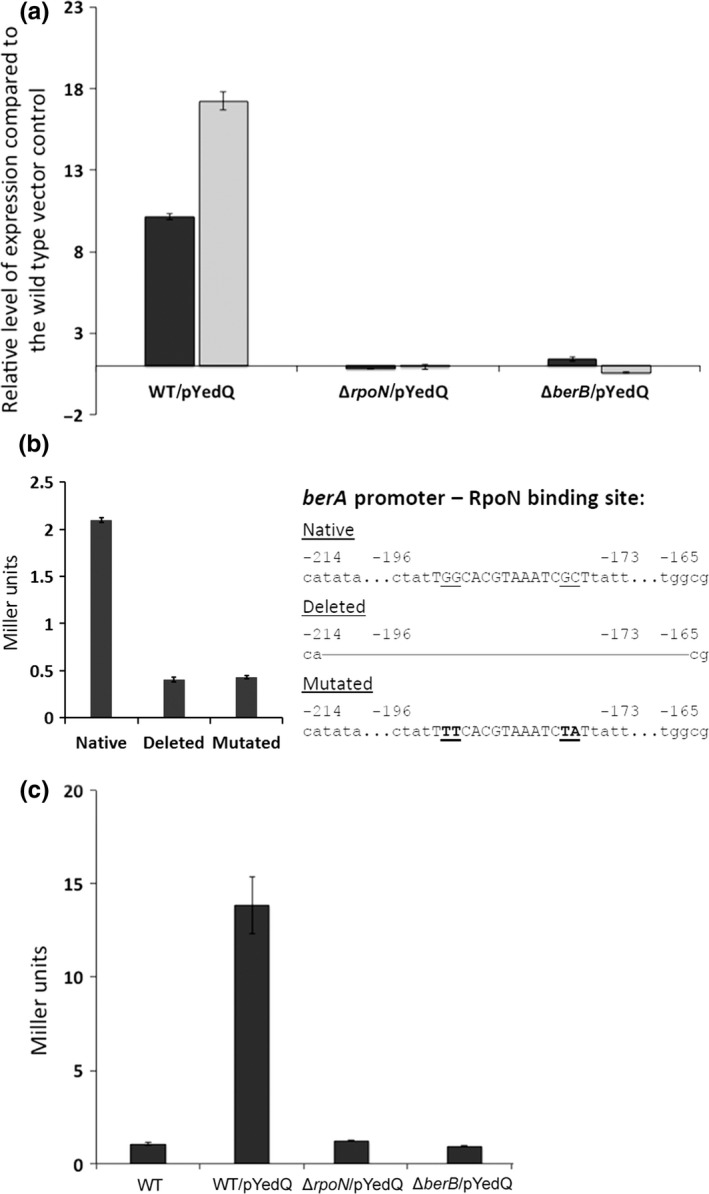
(a) QRT‐PCR analysis of transcript levels of *berA* (black columns) and *bepB* (gray columns) genes in c‐di‐GMP‐overproducing strains of the wild‐type (WT/pYedQ), Δ*rpoN* (Δ*rpoN*/pYedQ), and Δ*berB* (Δ*berB*/pYedQ). Data are normalized to *gyrB* transcript levels and presented as the fold change with respect to the wild‐type vector control strain for each gene. (b) Determination of *berA* expression by measuring the β‐galactosidase activity in c‐di‐GMP‐overproducing (pYedQ2‐containing) wild‐type bacteria harboring miniTn7‐inserted *berA*::lacZ promoter fusions with a native, deleted or mutated RpoN‐binding site. The schematics display the native, deleted and mutated RpoN‐binding site. (c) Determination of *bepB* expression by measuring the β‐galactosidase activity in c‐di‐GMP‐overproducing *bepB*::lacZ reporter strains of the wild‐type (WT/pYedQ), Δ*rpoN* (Δ*rpoN*/pYedQ), and Δ*berB* (Δ*berB*/pYedQ). In all cases (a, b, and c), data are representative of three independent biological experiments, and bars indicate standard deviations

We previously showed that overproduction of c‐di‐GMP or the BerA protein in the wild‐type strain causes the formation of wrinkled colonies on solid medium (Fazli et al., 2013). In the present study, we found that Δ*rpoN* and Δ*berB* strains with increased c‐di‐GMP levels do not form wrinkled colonies (Figure 1). In contrast, overproducing BerA from a plasmid in the Δ*rpoN* and Δ*berB* strains resulted in formation of wrinkled colonies (Figure 5). These findings suggest that BerA is crucial for wrinkled colony formation, and that the Δ*rpoN* and Δ*berB* strains with high c‐di‐GMP levels lack sufficient amounts of BerA protein required to form wrinkled colonies. It also indicates that RpoN and BerB exert their regulatory effect upstream of BerA. To test this further, we carried out qRT‐PCR analyses and observed an induction of *berA* transcription in response to high c‐di‐GMP levels, which was dependent on the presence of both the *rpoN* and *berB* genes. The high c‐di‐GMP levels in the pYedQ‐containing wild‐type strain caused a 10‐fold increased level of the *berA* transcripts compared with the vector control strain (Figure 6a). In contrast, we saw no significant increase in *berA* transcript levels in the pYedQ‐containing Δ*rpoN* and Δ*berB* strains (Figure 6a). We also measured β‐galactosidase activity in c‐di‐GMP‐overproducing (pYedQ2‐containing) bacteria harboring a promoterless *lacZ* gene fused to the *berA* promoter with or without mutated putative RpoN‐binding sites. Unlike the native *berA*‐*lacZ* fusion, the *berA*‐*lacZ* fusions with a mutated or deleted RpoN‐binding site were only expressed at a low level (Figure 6b).

In a previous study (Fazli et al., 2013), we showed that overproduction of c‐di‐GMP or BerA in the wild‐type strain leads to increased transcription of the exopolysaccharide biosynthesis gene *bepB* (Bcam1331). In the present study, we examined the involvement of *rpoN* and *berB* on activation of *bepB* transcription in response to high levels of c‐di‐GMP. Contrary to the results obtained with the wild‐type, we observed no significant increase in *bepB* transcript levels when c‐di‐GMP levels were elevated in the Δ*rpoN* and Δ*berB* strains (Figure 6a). We also measured β‐galactosidase activity from a chromosomally encoded reporter in which a promoterless *lacZ* gene together with a ribosome‐binding site was fused to the *bepB* gene. β‐Galactosidase activity was increased when c‐di‐GMP levels were elevated in the wild‐type strain but not in the Δ*rpoN* and Δ*berB* strains (Figure 6c). Taken together, these findings indicate that RpoN and BerB control the transcription of *berA* in response to high cellular c‐di‐GMP levels.

### 3.6 BerB binds to *berA* promoter DNA and interacts with RpoN

**Figure 7:**
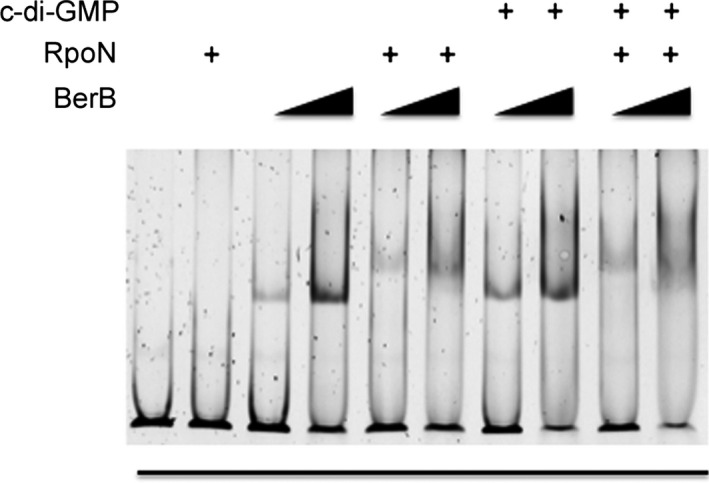
Binding of BerB and RpoN to the promoter region of the *berA* gene assessed by the use of EMSA. The RpoN and BerB proteins, and c‐di‐GMP were added into the EMSA reaction mixtures as indicated. EMSA, electrophoretic mobility shift assay

**Figure 8:**
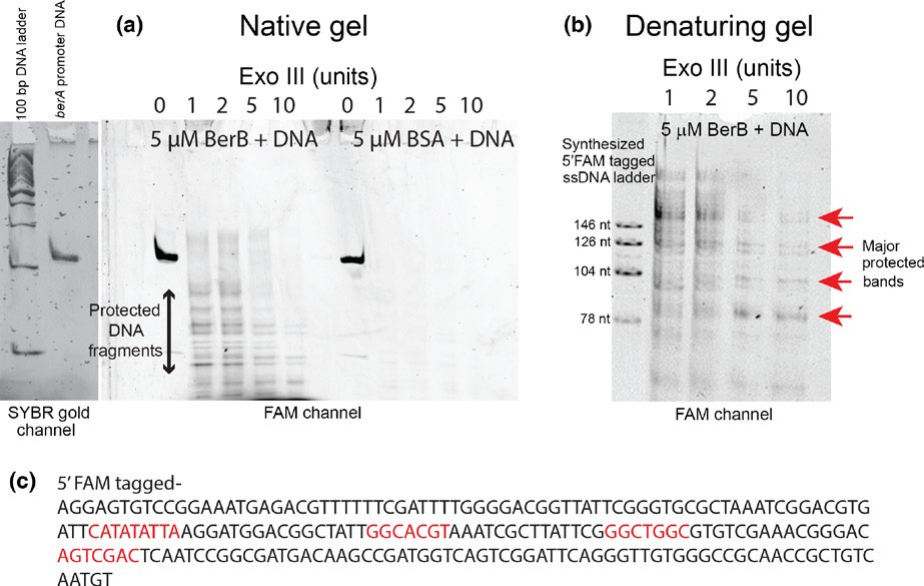
Mapping of BerB binding sites at the *berA* promoter region using exonuclease III footprinting. Protection of distinct DNA sequences by BerB, but not by BSA, was visualized by native gradient TBE gel electrophoresis (a). The size of the protected DNA fragments was estimated by denaturing Urea‐TBE electrophoresis with synthesized ssDNA ladders (b). Thereby putative BerB binding sites at the *berA* promoter region could be mapped as shown (c)

Most bEBPs bind to DNA at sites typically located ~80–150 bp upstream of the RpoN‐dependent promoters they control. To test if BerB directly binds to the *berA* promoter DNA, we carried out an electrophoretic mobility shift assay (EMSA) using purified BerB and a 216‐bp DNA fragment spanning −280 to −74 base pairs relative to the translational start of *berA*. BerB caused a shift in the mobility of the DNA fragment in a concentration‐dependent manner (Figure 7), indicating that BerB binds to the *berA* promoter DNA *in vitro*. We observed a substantial difference in *berA* transcript levels in the Δ*berB* strain compared with the wild‐type strain when the cellular c‐di‐GMP levels were elevated (Figure 6a). Hence, we hypothesized that c‐di‐GMP and BerB regulate *berA* transcription in concert and that c‐di‐GMP might have an effect on the DNA‐binding ability of BerB. However, the addition of c‐di‐GMP to the reaction mixtures did not change the DNA‐binding activity of BerB in our assay (Figure 7). The addition of RpoN to the reaction mixtures together with BerB shifted the mobility of the *berA* promoter DNA more than did BerB alone (Figure 7). We also tested the ability of RpoN to bind to the *berA* promoter DNA without BerB. In the absence of BerB, RpoN did not cause a detectable shift in the mobility of the *berA* promoter DNA fragment (Figure 7). Together, these results indicate that BerB and RpoN physically interact on the *berA* promoter DNA. Binding of RpoN to the *berA* promoter DNA in the absence of BerB may require the presence of the RNA polymerase. We subsequently investigated binding of BerB to the *berA* promoter region by the use of an exonuclease III footprinting assay (Figure 8). Unlike BSA, BerB was found to protect distinct sequences in the *berA* promoter region. The use of ad hoc synthesized FAM‐tagged ssDNA fragments allowed us to determine the size of the protected DNA fragments, and map putative BerB binding sites on the promoter DNA as shown in Figure 8.

### 3.7 BerB binds c‐di‐GMP

**Figure 9:**
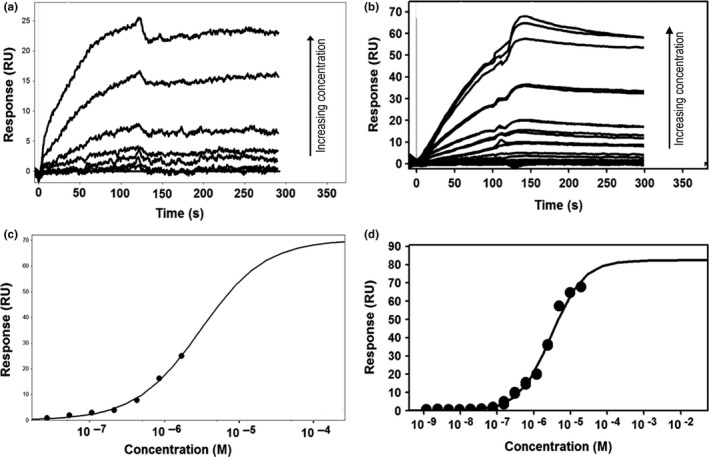
SPR sensorgrams indicating the binding of BerA (a) and BerB (b) to biotinylated c‐di‐GMP are depicted in the upper part of the figure. A range of concentrations was used for each protein. The signal heights in the steady state were taken to approximate the *K*_*D*_ values for each protein by scrubber software. The affinity fits for BerA (c) and BerB (d) are shown in the lower part of the figure. SPR, surface plasmon resonance

Our gene expression data demonstrated that RpoN and BerB induce *berA* transcription when the cellular c‐di‐GMP levels are high (Figure 6a). This made us hypothesize that BerB may sense the levels of c‐di‐GMP by directly binding to the signaling molecule. Accordingly, we investigated binding of BerB to c‐di‐GMP using SPR. As a positive control in our SPR assay, we included the BerA protein, which is a known c‐di‐GMP‐binding protein (Fazli et al., 2011). In accordance with our previous results (Fazli et al., 2011), BerA was found to bind the biotinylated c‐di‐GMP in a concentration‐dependent manner with an estimated *K*_*D*_ value of 3 μmol/L (Figure 9). Subsequently, we tested the purified BerB protein in different concentrations for its ability to bind c‐di‐GMP. We observed that BerB was able to bind to the biotinylated c‐di‐GMP in a concentration‐dependent manner with an estimated *K*_*D*_ value of approximately 3 µmol/L (Figure 9). The binding affinity of BerB falls within the physiological affinity range of previously characterized c‐di‐GMP‐binding proteins (Pultz et al., 2012). In addition to the SPR assay, binding of c‐di‐GMP to BerB was also demonstrated by the use of a differential scanning fluorimetry (DSF) assay. DSF measures the melting temperature of a protein, and exploits that the melting point of the protein may shift in the presence of small interacting ligands (Niesen, Berglund, & Vedadi, 2007). In the presence of increasing concentrations of c‐di‐GMP, we observed a shift in the melting temperature of BerB beginning at 1 μmol/L c‐di‐GMP, and increasing up to around 10 μmol/L c‐di‐GMP (Figure S3). These data indicate a binding of c‐di‐GMP to BerB with a *K*_*D*_ value between 1 and 10 μmol/L. Together, the SPR and DSF experiments suggest that BerB is a c‐di‐GMP‐binding protein.

## 4 Discussion

In contrast to other alternative sigma factors, RpoN is widely represented in the bacterial kingdom (Merrick, 1993). It was originally identified as a sigma factor required for transcription of the genes involved in nitrogen assimilation (Hirschman, Wong, Sei, Keener, & Kustu, 1985; Reitzer & Schneider, 2001). However, a great deal of evidence has now accumulated that RpoN also controls many other biological activities in bacteria, ranging from utilization of alternative carbon sources (Lundgren, Harris, Serwar, Scheel, & Nomura, 2015) and biodegradation of pollutants (Shingler, 2003) to motility (Dasgupta et al., 2003; Saldias, Lamothe, Wu, & Valvano, 2008; Totten, Lara, & Lory, 1990), and biofilm formation (Saldias et al., 2008; Thompson, Webb, Rice, & Kjelleberg, 2003; Wolfe, Millikan, Campbell, & Visick, 2004). It has been shown that RpoN controls flagellum‐mediated motility in *B. cenocepacia*, which was found to be essential for biofilm formation in microtiter trays (Saldias et al., 2008). However, the Δ*rpoN* strain still possessed intact flagella on its surface, indicating that, unlike in other bacteria, RpoN does not regulate flagellum biosynthesis in *B. cenocepacia* (Lardi et al., 2015; Saldias et al., 2008). In a recent study, it was shown that RpoN positively regulates expression of the genes involved in biosynthesis of the *Burkholderia* exopolysaccharide cepacian under nitrogen‐limiting conditions (Lardi et al., 2015). Furthermore, RpoN was found to regulate the expression of the *bapA* gene, coding for a large surface protein previously shown to be important for biofilm formation in *B. cenocepacia* (Inhülsen et al., 2012). In accordance with these previous studies, our flow‐cell experiments indicate that *B. cenocepacia* Δ*rpoN* strains have biofilm formation defects.

Here, we provide evidence that RpoN and the putative bEBP BerB regulate expression of the *berA* gene, coding for a c‐di‐GMP‐responsive transcriptional regulator previously shown to activate the expression of *bep* exopolysaccharide biosynthesis genes in *B. cenocepacia* (Fazli et al., 2013). We identified a putative RpoN‐binding site in the promoter region of the *berA* gene, and sequence analysis of the *berB* gene, located in the vicinity of *berA*, suggested that it encodes an RpoN‐interacting transcriptional regulator. Accordingly, our gene expression data demonstrated that both *rpoN* and *berB* are required for the expression of *berA* and, by extension, *bep* exopolysaccharide genes under high cellular c‐di‐GMP conditions. Evidence has been provided that Bep exopolysaccharide is important for the stability of flow‐cell grown *B. cenocepacia* biofilms, and for the formation of wrinkled colonies on solid medium in response to high levels of c‐di‐GMP (Fazli et al., 2013). Our flow‐cell biofilm experiments indicated that the Δ*berB* strain forms biofilm with reduced stability as previously reported for the Δ*berA* strain (Fazli et al., 2011) and Δ*bep* exopolysaccharide‐mutant strains (Fazli et al., 2013). Moreover, contrary to the wild‐type, but similar to what has previously been reported for the Δ*berA* (Fazli et al., 2011) and Δ*bep* strains (Fazli et al., 2013), the Δ*berB* strain did not form wrinkly colonies in response to high levels of c‐di‐GMP.

The domain architecture of BerB and the presence of the signature GAFTGA motif, essential for interaction with RpoN, indicate that BerB is a bEBP. We provide evidence that BerB directly binds to c‐di‐GMP, and our data further indicate that this binding is important for c‐di‐GMP‐mediated induction of *berA* transcription and exopolysaccharide production in *B. cenocepacia*. Several bEBPs have been shown to activate transcription of genes involved in exopolysaccharide production in other bacteria including *Pseudomonas aeruginosa* and *Vibrio* species. In *Vibrio fischeri*, the bEBP‐type response regulator SypG controls the transcription of symbiosis polysaccharide (Syp) genes and biofilm formation in a manner dependent on RpoN (Visick, 2009; Yip, Grublesky, Hussa, & Visick, 2005). Whether SypG is able to bind to c‐di‐GMP is not known. In contrast, the bEBP‐type transcriptional regulator VpsR from *Vibrio cholerae* controls expression of *Vibrio* polysaccharide (Vps) genes and biofilm formation independent of RpoN (Yildiz, Dalganov, & Schoolnik, 2001). VpsR binds to c‐di‐GMP and in turn activates the transcription of the *vpsT* gene (Srivastava, Harris, & Waters, 2011), coding for a LuxR‐type transcriptional regulator required for the expression of Vps biosynthesis genes (Casper‐Lindley & Yildiz, 2004). In *P. aeruginosa*, the c‐di‐GMP‐binding protein FleQ is an unusually versatile bEBP. While the regulation of flagella biosynthesis genes by FleQ depends on RpoN (Dasgupta et al., 2003; Jyot, Dasgupta, & Ramphal, 2002), FleQ controls the expression of the Pel polysaccharide genes independent of RpoN in response to changing cellular c‐di‐GMP levels (Baraquet, Murakami, Parsek, & Harwood, 2012; Hickman & Harwood, 2008). It was initially demonstrated that FleQ binds to the *pel* promoter DNA and represses *pel* transcription in the absence of c‐di‐GMP. Upon c‐di‐GMP binding to FleQ, this inhibition is relieved and the transcription from the *pel* promoter is activated (Hickman & Harwood, 2008). However, it was later found that, in the presence of c‐di‐GMP, FleQ also functions as an activator when bound to a second site on the *pel* promoter DNA (Baraquet et al., 2012), which indicates that c‐di‐GMP converts FleQ from a repressor to an activator of exopolysaccharide gene transcription. Our data suggest that BerB activates gene transcription upon c‐di‐GMP binding, and unlike VpsR and FleQ, requires RpoN. BerB therefore represents the first example of a bEBP that functions together with both RpoN and c‐di‐GMP.

We showed that BerB binds to the *berA* promoter DNA in vitro. However, the addition of c‐di‐GMP did not change the DNA‐binding activity of BerB, suggesting that binding of c‐di‐GMP may instead have effects on other functions of BerB such as its ATPase activity. Recent studies have suggested that c‐di‐GMP binds to AAA+ ATPase‐domain containing proteins from diverse bacteria and, except for only one case, inhibits their ATPase activity (Baraquet & Harwood, 2013; Roelofs et al., 2015; Trampari et al., 2015). For example, c‐di‐GMP competes with ATP to bind to the ATP‐binding site on FleQ and inhibits FleQ′s ATPase activity (Baraquet & Harwood, 2013), but in the case of MshE from *V. cholerae*, binding of c‐di‐GMP to the N‐terminal domain of MshE enhances its ATPase activity (Roelofs et al., 2015). In our case, BerB appears to activate RpoN‐dependent transcription of the *berA* gene. Hence, if c‐di‐GMP binding has an effect on BerB's ATPase activity, then it should be stimulation rather than inhibition, possibly by affecting the oligomerization of the AAA+ domain on BerB, which is essential for the ability of bEBPs to hydrolyze ATP to drive the RpoN‐RNA polymerase closed complex into a transcriptionally active open complex. Alternatively, c‐di‐GMP may affect BerB's interaction with RpoN.

**Figure 10:**
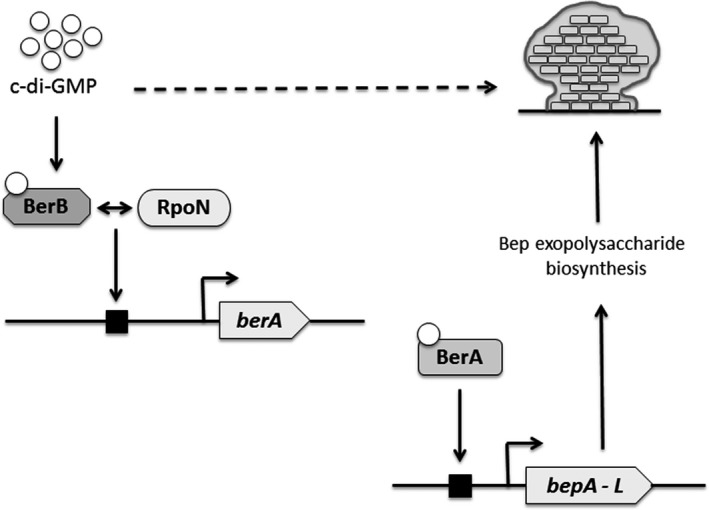
Model for the regulation of biofilm formation by c‐di‐GMP, BerB, and RpoN. BerB binds c‐di‐GMP and activates RpoN‐dependent transcription of the *berA* gene coding for the c‐di‐GMP‐binding transcriptional regulator BerA. An increased level of the BerA protein in turn induces the production of biofilm‐stabilizing Bep exopolysaccharide in response to high c‐di‐GMP levels

Exopolysaccharides are a major component of the biofilm matrix with importance for the mechanical stability of biofilms. However, their biosynthesis is an energy‐intensive process and therefore requires tight regulation. The requirement of an activator protein by the RpoN‐RNA polymerase closed complex to initiate transcription of genes related to exopolysaccharide biosynthesis, such as the regulation of *berA* and by extension *bep* genes described here, allows tight control of gene expression in the off‐state until the activating signal is present. Furthermore, our work suggests that c‐di‐GMP regulates Bep exopolysaccharide production in *B. cenocepacia* both by stimulating *berA* transcription and by stimulating transcriptional activation by BerA. This results in a cascade regulation of a biofilm exopolysaccharide in which two consecutive transcription events are both activated by c‐di‐GMP (Figure 10). Such sustained c‐di‐GMP‐mediated regulation may allow tight control of expenditure of cellular resources. The BerB protein represents the first example of a bEBP, whose regulatory function depends on both RpoN and c‐di‐GMP. However, mechanistic questions relating to how c‐di‐GMP modulates BerB‐RpoN‐dependent transcription of the *berA* gene remain to be answered.

## Conflict of Interest

None declared.
