# Supplementary material for: Regulation of Burkholderia cenocepacia biofilm formation by RpoN and the c‐di‐GMP effector BerB
Source: Microbiologyopen. 2017 Apr 16;6(4):e00480. doi: 10.1002/mbo3.480 (PMC5552954; doi:10.1002/mbo3.480)
Supplement: Supplementary file 1 [file MBO3-6-na-s001.pdf]

Supplemental material

Regulation of *Burkholderia cenocepacia* biofilm formation by RpoN and the c-di-GMP effector BerB

Mustafa Fazli, Morten Rybtke, Elisabeth Steiner, Elisabeth Weidel, Jens Berthelsen, Julie Groizeleau, Wu Bin, Boo Zhao Zhi, Zhang Yaming, Volkhard Kaever, Michael Givskov, Rolf W. Hartmann, Leo Eberl, and Tim Tolker-Nielsen

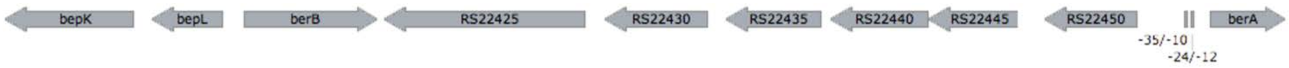

Figure S1. Map of the *B. cenocepacia* genomic region containing the *berA* and *berB* genes, the genes in between, and the last two genes (*bepK* and *bepL*) of the *bepA-L* cluster. The -35/-10 and -24/-12 promoter elements are also shown. The genes are not drawn to scale.

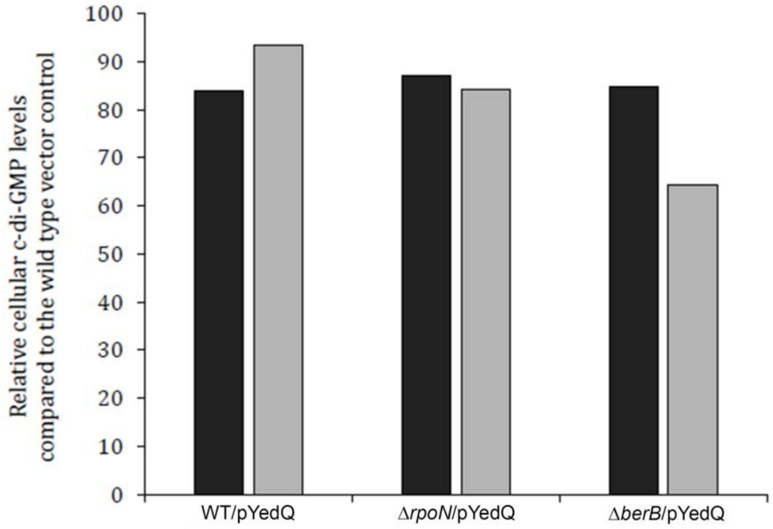

Figure S2. Cellular c-di-GMP levels of the wild type (WT), and the *ΔrpoN* and *ΔberB* strains carrying pYedQ relative to the wild type vector control strain. Black columns, culture grown cells; gray columns, biofilm grown cells. c-di-GMP was extracted from biofilm grown *B. cenocepacia* strains on membrane filters placed on AB medium supplemented with 2% agar (w/v), 0.2% glucose (w/v), 0.1% casaminoacids (w/v) for 48 hours. The cells were washed off the filters with physiological saline and harvested by centrifugation. Alternatively, the strains were grown in AB medium supplemented with 0.2% glucose, 0.1% casaminoacids to an OD600 of approximately 1.5, and 5 ml of the cell cultures were harvested. c-di-GMP was extracted as described previously (Spangler et al., 2010) with minor modifications. The cell pellets were resuspended in 300 µl of ice-cold extraction solvent (acetonitrile/methanol/water, 40/40/20, v/v/v) and incubated on ice for 15 min. The cell suspension was then heated to 95°C for 10 min. After cooling on ice, the suspension was centrifuged at maximum speed (16.000 rcf) for 10 min. The extraction of the remaining pellet was repeated twice with 200 µl of extraction solvent at 4°C. The solvent of the combined supernatants was removed in a Speedvac at 60°C. Quantification of c-di-GMP was performed by HPLC-coupled tandem mass spectrometry as previously described (Burhenne and Kaever, 2013) with the exception that an LC-10AD VP HPLC system (Shimadzu) and an API4000 mass spectrometer (Sciex) were used.

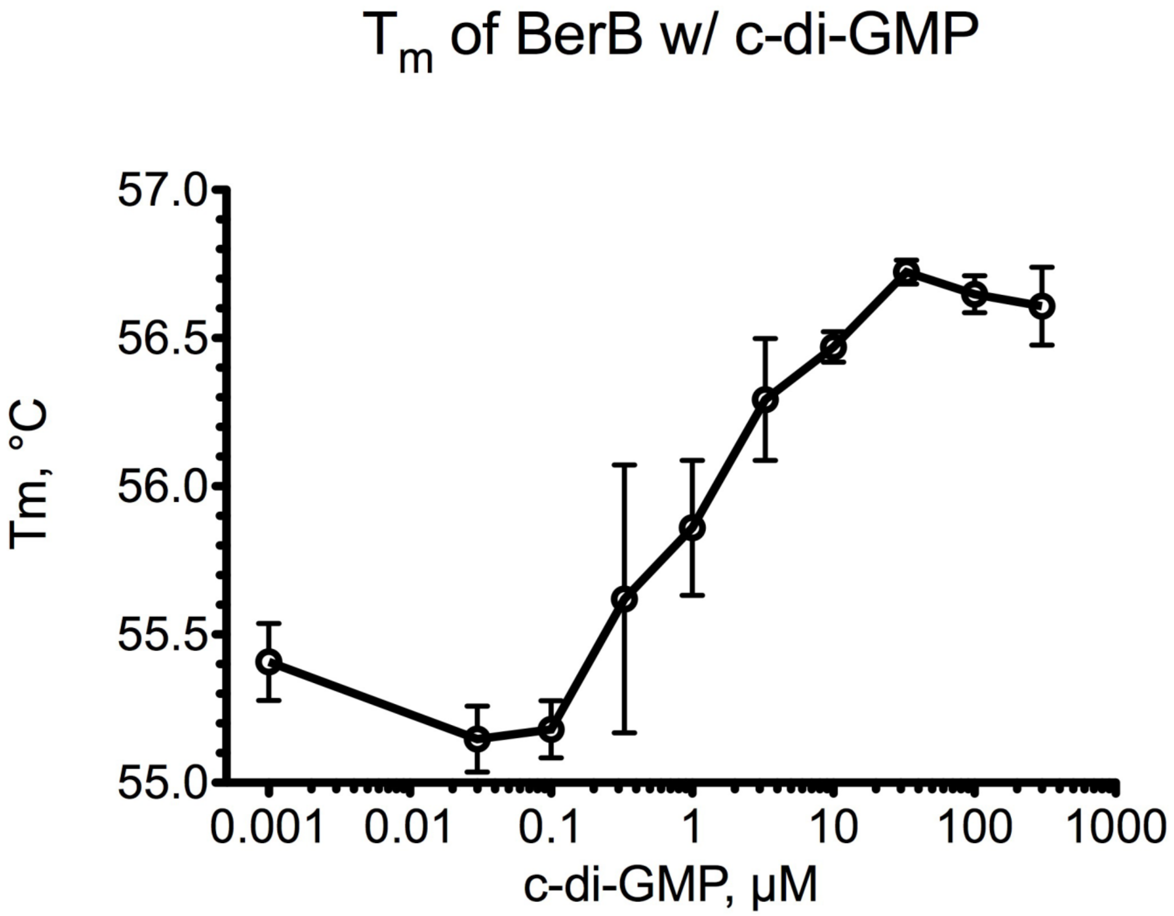

Figure S3. Binding of c-di-GMP to BerB demonstrated by the use of a differential scanning fluorimetry assay. A LightCycler 480 Real-Time PCR System (Roche Applied Science) was used for the thermal denaturation experiments. The reactions were carried out in a 384 well plate in 10 µl reaction volumes. BerB protein at a final concentration of 1 µM in sample buffer (50 mM HEPES (pH 7.5), 150 mM NaCl, 1 mM DTT, and 1:1000 dilution of SyproOrange 5000X (Invitrogen)) was mixed on ice with increasing concentrations of c–di-GMP in sample buffer as indicated. The plate was transferred to the Lightcycler 480, and the temperature was increased stepwise 3.4 °C/min from 20 °C to 95 °C. Tm shifts were calculated by the use of a LightCycler Tm Calling analysis software module. Each point represents the average of 5 independent measurements. The standard deviation for each point is indicated by vertical bars.

References:

Burhenne, H., and Kaever, V. (2013) Quantification of cyclic dinucleotides by reversed-phase LC-MS/MS. Methods Mol Biol. 1016:27-37.

Spangler, C., Böhm, A., Jenal, U., Seifert, R., and Kaever, V. (2010) A liquid chromatography-coupled tandem mass spectrometry method for quantification of cyclic di-guanosine monophosphate. J Microbiol Methods. 81:226-31.
